# Evidence-based application of explicit criteria to assess the appropriateness of geriatric prescriptions at admission and hospital stay

**DOI:** 10.1371/journal.pone.0238064

**Published:** 2020-08-25

**Authors:** Enrica Di Martino, Alessio Provenzani, Piera Polidori

**Affiliations:** Clinical Pharmacy Service, Mediterranean Institute for Transplantation and Advanced Specialized Therapies (ISMETT), Palermo, Italy; University of Toronto, CANADA

## Abstract

**Background:**

Inappropriate prescribing in the elderly is a critical issue in primary care, causing a higher risk of Adverse Drug Reactions (ADRs) and resulting in major patient safety concerns. At international level, many tools have been developed to identify Potentially Inappropriate Medications (PIMs).

**Objective:**

The aim of this study was the application of Beers, Screening Tool of Older People's Prescriptions (STOPP)/Screening Tool to Alert to Right Treatment (START) and Improving Prescribing in the Elderly Tool (IPET) criteria as key tool to improve the quality of prescribing.

**Methods:**

A retrospective study was conducted using the aforementioned criteria. Two different cohorts of elderly patients were enrolled between January 2015 and December 2016, 1800 at admission and 1466 at hospital stay. The index of each criterion divided by politherapy were correlated with comorbidities (Pearson correlation). A comparison was made between admission and hospital stay through a Student's *t* test of the average of the index.

**Results:**

The Proton Pump Inhibitors (PPIs) were the most prescribed PIMs according Beers criteria in both patient cohorts (56%). The most detected drug-drug and drug-disease interactions at admission and at hospital stay were 3 or more drugs active on the Central Nervous System (CNS) as they can predispose to fall-risk. The most detected PIMs with STOPP criteria at admission were PPIs administered for more than 8 weeks. Inhaled β2-agonists or antimuscarinics were the most prescribed Potential Prescription Omissions (PPOs) according to START criteria. Nonsteroidal Anti-inflammatory Drugs (NSAIDs) in patients with high blood pressure were the most detected PIMs according to IPET criteria during hospital stay. A significant correlation between the comorbidities and the all index at hospital stay, while at admission there was no significant correlation for Beers and IPET index.

**Conclusion:**

The prescriptive criteria were a useful tool for assessing the quality of prescriptions in the geriatric population and identifying their critical issues.

## Introduction

In recent decades, the demographic change in the population has resulted in an increase in life expectancy with a consequent rapid aging of the population worldwide. According to the World Health Organization's World Report on Aging and Health [1], in 2050 the global population aged 60 years or over will increase from the current 900 million to almost 2 billion. This increase in life expectancy coincides with an increase in multiple chronic diseases and conditions of non-self-sufficiency that require long-term assistance, causing a strong impact on all health systems in industrialized countries.

Elderly patients, defined as patients over 65 years [2], are at increased risk of ADRs [3] due to age-related physiological changes with consequent alteration of the normal response to pharmacological therapy. These physiological changes are complex and depend on numerous factors, including the composition of the body mass, the health conditions of the various organs and the activity of the enzymatic systems [4–6].

All these conditions are emphasized by the use of multiple medications which makes it difficult to prescribe drugs with the right risk-benefit ratio [7]. The prescription of PIMs in the elderly is associated with an increased risk of ADR compared to the expected benefits of treatment, particularly when a safer or more effective alternative treatment is available for the same clinical condition. Inappropriate prescribing may be due to inappropriate dosage, incorrect duration of treatment, contraindicated use, drug-drug or drug-disease interactions. [8,9]. A further aspect of inappropriate prescription is the PPO of appropriate medicines based on the patient's age or the fact that he is already taking too many drugs.

At international level, many explicit criteria have been developed as tools to identify PIMs and ensure the safety of prescriptions in the elderly. These criteria are based on scientific evidence through the standard consensus search methodologies (Delphi consensus), consequently they need periodic updates to ensure their reliability. These are lists of drugs or classes of drugs to be avoided or used with caution in elderly patients [10]. The most used explicit criteria are the Beers criteria [11], the STOPP/START criteria [12] and the IPET criteria [13].

In particular, the Beers criteria were developed by the American Geriatrics Society and include the PIMs to be avoided in older adults divided into 6 lists: drugs to be avoided in most elderly or elderly patients with specific diseases, drug-disease interactions, drug-drug interactions, drugs to be used with caution, drugs for which dose adjustment is required based on kidney function and drugs with strong anticholinergic properties [11]. The STOPP/START criteria were developed in Ireland and include PIMs (STOPP criteria) and PPOs (START criteria), both grouped by anatomical classes [12]. The IPET criteria consist of a list of 14 PIMs identified by a panel of Canadian experts [13].

The choice of one criterion over another is complex and depends on numerous factors, such as drugs availability in the market and legislative measures in each country. STOPP/START criteria, contrary to Beers and IPET criteria, take account of PPOs which may be just as important as PIMs in the overall assessment of drug appropriateness in the elderly. In this regard, it is useful to use all four criteria to detect a greater number of PIMs/PPOs and achieve more appropriate and safer therapies.

A possible implementation of these explicit criteria could be the algorithm-driven electronic medical record notification that enhances the detection of PIMs and PPOs during prescription, based on generalities of patient and taken drugs.

However, the evaluation of prescription appropriateness in the elderly represents a complex issue that should be based on clinical judgment and these criteria should be intended as a support tool in choosing the most appropriate drugs, avoiding those at greatest risk of ADR.

In this context, the aim of this study was to assess the medication use in elderly patients in terms of PIMs and PPOs applying Beers, STOPP/START and IPET criteria. In particular, a comparison was made between the PIMs and the PPOs detected at admission and those detected at the hospital stay.

## Materials and methods

A retrospective observational study was conducted by the Tracer Pharmacist (TP) in 24 months at the Clinical Pharmacy Service of the Mediterranean Institute for Transplantation and Advanced Specialised Therapies (ISMETT), a Research Institute in Palermo, Italy. This study was approved by the Institutional Review Board (n. 20/17) and the Ethic Committee (Protocol No. 001-178-GEN/2018) of ISMETT and written informed consent was obtained from all patients. The data processing of the patients enrolled in this study was done in compliance with the provisions and prescriptions of the policies and procedures adopted by ISMETT and the current legislation, also with regard to the security measures adopted for the protection of the aforementioned data. All patients aged ≥ 65 admitted to ISMETT between January 2015 and December 2016 were enrolled. Two different patient cohorts were analyzed, one relating to admission and one relating to hospital stay, and the respective information related to the clinical profile of patients and the PIMs or PPOs have been inserted into two specific databases.

In particular, for each patient, generalities such as age and gender, date of admission and discharge, diagnosis at admission and any comorbidities were detected. Clinical information such as the blood pressure, the international normalized ratio, the blood levels of potassium and sodium and the predisposing risk factors for falling were obtained through consultation of the electronic medical record Sunrise Clinical Manager (Eclipse) ®.

In order to assess the overall quality of drug prescribing in the elderly, four evidence-based criteria were used: the Beers criteria (2015 version), the STOPP/START criteria (2014 version) and the IPET criteria (2000 version). The Beers, STOPP and IPET criteria allow the detection of PIMs, while the START criteria allow the detection of PPOs.

All prescriptions once a day or of a duration not exceeding three days made during hospital stay were excluded from the analysis, because they were considered not clinically significant to justify each of the aforementioned criteria used.

### Statistical analysis

GraphPad Prism 8 ® software was used for statistical analysis. Values of p <0.05 were considered statistically significant. The statistical analysis involved the evaluation of the means and standard deviations for the parametric variables. As part of the retrospective study, the criteria of prescriptive inapropriateness (Beers, STOPP and IPET) and prescriptive appropriateness (START) were expressed in the form of Index (I) of the criteria with polytherapy.

These index are expressed by the formula:

$$I=\frac{N{}^{\circ}\;criteria\;(Beers,STOPP,START,IPET)}{TOT\;of\;taken\;drugs}$$

In order to identify the outliers relating to the index of the criteria, the statistical analysis was preceded by a data cleaning phase, in which it was calculated the z scores [14], given by the ratio between the deviation of the average of the index (I - Ī) for standard deviation (SD):

$$z=\frac{\left(I–Ī\right)}{SD}$$

Outliers with a z > | 3 | score have been replaced with a maximum score of +3. The resulting index were correlated with the comorbidities detected at both admission and hospitalization (Pearson *r* correlation). The comparison of the admission and hospital stay index averages was made by estimating Cohen's d effect size [15] and Student's *t* test.

## Results

Two different cohorts of elderly patients were enrolled in this study, 1800 at admission and 1466 at hospital stay. Characteristics of both cohorts of patients and the respective pathologies are shown in Tables 1 and 2. Table 1 shows an increase in the average consumption of drugs from admission to hospital stay (respectively from 5.39 ± 3.00 to 10.82 ± 6.09) in response to a concomitant increase in the number of registered pathologies (from 2.83 ± 1.58 to admission to 3.52 ± 2.12 to hospital stay).

**Table 1:** Patient characteristics at admission and hospitalization.

	Category	Admission	Hospital stay
TOT of enrolled patients	Number	1800	1466
Gender	males	1099 (61%)	890 (61%)
	females	701 (39%)	576 (39%)
Age (years)	M; SD	73.22 ± 5.83	73.12 ± 5.71
	65–70	692 (38%)	555 (38%)
	71–80	906 (50%)	739 (41%)
	81–90	190 (11%)	165 (9%)
	> 90	12 (1%)	7 (less than 1%)
Length of stay (days)	3–7	Not applicable	567 (39%)
	8–14		564 (38%)
	15–20		144 (10%)
	21–30		96 (7%)
	≥ 31		95 (6%)
Comorbidity	M; SD	2.83 ± 1.58	3.52 ± 2.12
	0–3	1201(67%)	741 (51%)
	4–6	579 (32%)	600 (41%)
	≥ 7	20 (1%)	125 (9%)
Taken drugs	M; SD	5.39 ± 3.00	10.82 ± 6.09
	1–3	553 (31%)	185 (13%)
	4–6	664 (37%)	187 (13%)
	7–9	400 (22%)	260 (18%)
	10–12	149 (8%)	283 (19%)
	13–16	34 (2%)	316 (22%)
	17–20		142 (10%)
	21–24		64 (4%)
	25–28		16 (1%)
	29–41		13 (1%)

**Table 2:** Diseases registered at admission and during hospital stay.

Comorbidity	Admission (n = 1800)	Hospital stay (n = 1466)
Hypertension	1202 (67%)	1084 (74%)
Heart disease	906 (50%)	807 (57%)
History of falls and fractures	712 (40%)	876 (60%)
Heart failure	670 (37%)	685 (47%)
Cancer	472 (26%)	438 (30%)
Diabetes mellitus	403 (22%)	690 (47%)
Gastrointestinal disease	340 (19%)	1400 (95%)
Liver disease	295 (16%)	902 (62%)
Benign prostatic hypertrophy	208 (12%)	404 (28%)
Thyroid and parathyroid disorders	163 (9%)	872 (59%)
Lung disease	163 (9%)	307 (21%)
Pancreas disease	136 (8%)	303 (21%)
Hyperuricemia	127 (7%)	345 (24%)
Anxiety	101 (6%)	373 (25%)
Atrial fibrillation	84 (5%)	476 (32%)
Osteoporosis	77 (4%)	276 (19%)
Depression	77 (4%)	303 (21%)
Anemia	77 (4%)	347 (24%)
Chronic renal failure	55 (3%)	775 (39%)
Delirium/schizophrenia	35 (2%)	278 (19%)
Parkinson	17 (1%)	264 (18%)
Post-transplant follow-up	11 (1%)	38 (3%)
Systemic infection	9 (1%)	NA
Alzheimer/dementia	NA	1400 (35%)
Delirium/schizophrenia	NA	278 (19%)
Metabolic syndrome	NA	271 (18%)
Stroke	NA	270 (18%)
Neurological disorders	NA	261 (18%)

The wording NA (Not Applicable) corresponds to pathologies found upon admission but not to hospitalization, or vice versa.

Hypertension, heart disease and history of falls and fractures were the most common comorbidities in both cohorts of patients (Table 2).

As shown in the Table 3, during the hospital stay an increase of patients with PIMs was observed according to the Beers and IPET criteria, while they were reduced using the STOPP criteria. The START criteria, which instead concern the PPOs, are increased during hospitalization.

**Table 3:** PIMs and PPOs detection at admission and at hospital stay.

	Category	Admission	Hospital stay
Beers	% of patients with PIMs	77% (1393/1800)	89% (1244/1466)
	M; SD of PIMs	1.51 ± 1.34	2.64 ±1.86
	M; SD of I_Beers_ (Beers/tot.drugs)	0.29 ± 0.29	0.26 ± 0.19
	TOT detected PIMs	2720	3882
	• PIMs	56% (1512/2710)	56% (2186/3882)
	• Drugs to use with caution	32% (876/2720)	23% (888/3882)
	• Drug-drug interactions	5% (132/2720)	5% (295/3882)
	• Drug-disease interactions	4% (112/2720)	13% (516/3882)
	• Drug dose adjustment based on kidney function	2% (58/2720)	2% (84/3882)
	• Anticholinergic drugs	1% (30/2720)	less than 1% (9/3882)
STOPP	% of patients with PIMs	86% (1543/1800)	85% (1244/1466)
	M; SD of PIMs	7.78 ± 1.27	2.54 ± 1.79
	M; SD of I_STOPP_ (STOPP/tot.drugs)	0.36 ± 0.29	0.24 ± 0.19
	TOT detected PIMs	3216	3734
START	% of patients with PPOs	72% (1288/1800)	77% (1130/1466)
	M; SD of PPOs	1.52 ± 1.32	2.25 ± 1.88
	M; SD of I_START_ (START/tot.drugs)	0.023 ± 0.089	0.016 ± 0.048
	TOT detected PPOs	2729	3305
IPET	% of patients with PIMs	11% (198/1800)	17% (250/1466)
	M; SD of PIMs	0.11 ± 1.32	0.18 ± 0.43
	M; SD of I_IPET_ (IPET/tot.drugs)	0.28 ± 0.26	0.19 ± 0.16
	TOT detected PIMs	205	273

According to the Beers criteria, the type of PIMs most detected in both patient cohorts (56%) were the potentially inappropriate drugs, of which the PPIs were the most prescribed (Table 4). The most detected drug-drug and drug-disease interactions according to the Beers criteria both at admission and at hospital stay were, instead, 3 or more drugs active on the CNS as they can predispose to risk of falling.

**Table 4:** PIMs detected according to Beers criteria both at admission and hospitalization.

BEERS criteria		Admission	Hospital stay
Total of detected PIMs		n = 2720	n = 3882
**Drugs to be avoided in most elderly or elderly patients with specific diseases**		**n = 1512 (56%)**	**n = 2186 (56%)**
Proton-Pump Inhibitors (PPIs)		1022 (67%)	1145 (52%)
Peripheral alpha-1 blockers		103 (7%)	73 (3%)
Insulin, sliding scale		100 (7%)	342 (16%)
Short-acting benzodiazepines		85 (6%)	NA
Metoclopramide		77 (5%)	151 (7%)
Digoxin in patients with decreased renal clearance		47 (3%)	46 (2%)
Antidepressants, alone or in combination		23 (1%)	NA
Sulfonylureas		15 (1%)	NA
Digoxin in patients with atrial fibrillation		14 (1%)	40 (2%)
NSAIDs		10 (1%)	154 (7%)
Non-benzodiazepine sedative hypnotics		8 (1%)	18 (1%)
Centrally acting antihypertensives		NA	83 (4%)
Benzodiazepines with short and intermediate duration of action		NA	79 (4%)
Other PIMs that individually were not quantitatively relevant.		NA	55 (3%)
**Drugs to be used with caution**		**n = 876 (32%)**	**n = 888 (23%)**
Diuretics		714 (82%)	776 (87%)
Aspirin for primary prevention of cardiac events in adults aged 80 and over		70 (8%)	76 (9%)
SSRIs		53 (6%)	29 (3%)
Oxcarbazepine or carbamazepine		19 (2%)	NA
SNRIs		10 (1%)	NA
Tricyclic antidepressant		10 (1%)	NA
Antineoplastics such as carboplatin, cisplatin, cyclophosphamide and vincristine		NA	5 (1%)
Prasugrel in patients aged 75 and over		NA	less than 1%
**Drug-drug interactions**		**n = 132 (5%)**	**n = 199 (5%)**
Benzodiazepines and non-benzodiazepine sedative hypnotics	≥ 2 other CNS-active drugs	44 (33%)	28% (56)
Alpha-1 blockers	Loop diuretics	29 (22%)	16 (8%)
Antidepressants	≥ 2 other CNS-active drugs	28 (21%)	18 (9%)
Antipsychotics	≥ 2 other CNS-active drugs	19 (14%)	16 (8%)
Opioids	≥ 2 other CNS-active drugs	5 (4%)	81 (41%)
Warfarin	Amiodarone	4 (3%)	2 (1%)
ACEIs	Amiloride	1 (1%)	2 (1%)
Anticholinergics	Anticholinergics	1 (1%)	2 (1%)
warfarin	NSAIDs	1 (1%)	NA
Oral corticosteroids	NSAIDs	NA	8 (4%)
**Drug-disease interactions**		**n = 112 (4%)**	**n = 516 (13%)**
Antipsychotics	History of falls or fractures	39 (35%)	20 (4%)
Tricyclic antidepressant	History of falls or fractures	26 (23%)	NA
SSRIs	History of falls or fractures	19 (17%)	17 (3%)
Anticonvulsants	History of falls or fractures	12 (11%)	6 (1%)
Verapamil	Heart failure	4 (3%)	3 (1%)
Benzodiazepines	History of falls or fractures	3 (3%)	71 (14%)
Diltiazem	Heart failure	2 (2%)	NA
Non-benzodiazepine sedative hypnotics	History of falls or fractures	2 (2%)	10 (2%)
Opioids	History of falls or fractures	2 (2%)	369 (72%)
Thiazolidinediones	Heart failure	1 (1%)	NA
Tramadol	Chronic seizures or epilepsy	NA	5 (1%)
Anticholinergics	Benign prostatic hypertrophy and pathologies of the lower urinary tract	NA	4 (1%)
Antipsychotics	Dementia or cognitive impairment	NA	3 (1%)
Other PIMs that were not quantitatively relevant		NA	8 (2%)
**Drugs for which dose adjustment is required based on kidney function**			
**drugs**	**ClCr (ml/min)**	**n = 58 (2%)**	**n = 84 (2%)**
Enoxaparin	< 30	30 (52%)	41 (49%)
Pregabalin	< 60	10 (17%)	7 (8%)
Ranitidine	< 50	9 (15%)	8 (10%)
Spironolactone	< 30	3 (5%)	16 (19%)
Duloxetine	< 60	2 (3%)	1 (1%)
Amiloride	< 30	1 (2%)	NA
Rivaroxaban	30–50	1 (2%)	NA
Gabapentin	≤ 60	1 (2%)	9 (11%)
Pregabalin	< 30	1 (2%)	NA
Apixaban	< 25	NA	2 (2%)
**Drugs with strong anticholinergic properties**		**n = 30 (1%)**	**n = 9 (less than 1%)**
Paroxetine		11 (37%)	5 (56%)
Amitriptyline		7 (23%)	1 (11%)
Hydroxyzine		5 (17%)	NA
Olanzapine		2 (7%)	1 (11%)
Atropine (excludes ophthalmic)		2 (7%)	NA
Clozapine		1 (3%)	NA
Belladonna alkaloids		1 (3%)	NA
Scopolamine (excludes ophthalmic)		1 (3%)	NA
Chlorpheniramine		NA	2 (22%)

The wording NA (Not Applicable) corresponds to PIMs detected at admission but not at hospitalization, or vice versa.

On the other hand, the PIMs to be used with great caution in elderly patients according to Beers' criteria decreased from 32% to admission to 23% to hospitalization; diuretics were the most prescribed drugs as they can cause or exacerbate the Syndrome of Inappropriate Antidiuretic Hormone secretion (SIADH). PIMs based on kidney function and PIMs with strong anticholinergic properties according to Beers' criteria remained almost unchanged, with low percentages both at admission and during hospitalization (respectively 2% and 1%). In particular, the PIMs most found were enoxaparin in patients with creatinine clearance less than 30 mL/min and paroxetine as anticholinergic drug.

Regarding STOPP criteria, there was a reduction in PIMs at hospital stay compared to admission (86% and 85% respectively). As can be seen in Table 5, the PIMs most detected with the STOPP criteria have been PPIs for more than 8 weeks at admission, while at the hospital stay the vasodilators in patients with orthostatic hypotension and the drugs that can cause constipation were the most detected PIMs.

**Table 5:** PIMs detected with STOPP criteria and PPOs detected with START criteria both at admission and hospitalization.

PIMs detected with STOPP criteria	Admission	Hospital stay
**Total of detected PIMs**	**n = 3216**	**n = 3734**
Proton pump inhibitors for more than 8 weeks.	956 (30%)	NA
Vasodilators (α1-antagonists, Ca-antagonists, long-acting nitrates, ACEIs, ARBs, diazoxide, minoxidil, hydralazine) in patients with persistent orthostatic hypotension: increased risk of falls.	912 (28%)	765 (20%)
α1-antagonists in patients with symptomatic orthostatic hypotension or urination syncope (risk of precipitation of recurrent syncopes).	277 (9%)	166 (4%)
Benzodiazepines (increased risk of falls).	10 5 (3%)	109 (3%)
β-blockers in patients with symptomatic bradycardia (<50/min) as it can predispose to type II cardiac arrest or complete cardiac arrest.	95 (3%)	80 (2%)
Loop diuretics as first-line treatment of hypertension (lack of data on the results for this indication, safer and more effective alternatives available).	92 (3%)	121 (3%)
Aspirin associated with clopidogrel as prevention of secondary stroke unless the patient has inserted a coronary stent in the previous 12 months or a concomitant acute coronary syndrome or has symptomatic high-grade karyotid arterial stenosis (no evidence of additional benefit compared to monotherapy with clopidogrel).	87 (3%)	85 (2%)
Drugs that can cause constipation (anticholinergics, oral iron, opioids, verapamil, aluminum antacids) in patients with chronic constipation where the therapeutic alternatives are appropriate (risk of exacerbation of constipation).	86 (3%)	740 (20%)
Benzodiazepines for more than 4 weeks (risk of prolonged sedation, confusion, impaired balance, falls, traffic accidents; all benzodiazepines should be gradually reduced to avoid abrupt benzodiazepine withdrawal syndrome).	85 (3%)	24 (1%)
Opioids for protracted periods without concomitant laxative therapy.	65 (2%)	449 (12%)
Potassium-sparing diuretics with concomitant hyperkalemic drugs (ACEIs, amiloride, triamterene).	56 (2%)	248 (7%)
Digoxin in patients with heart failure with preserved ventricular function (no clear evidence of benefit).	55 (2%)	
Aspirin, clopidogrel, dipyridamole, vitamin K antagonists, direct thrombin inhibitors or factor Xa inhibitors in patients with risk of significant bleeding, severe uncontrolled hypertension, hemorrhagic diathesis (high risk of bleeding).	42 (1%)	124 (3%)
Oral doses of elemental iron greater than 200 mg/day (no evidence of higher absorption compared to lower dosages).	41 (1%)	77 (2%)
Amiodarone as the first line in supraventricular tachyarrhythmias.	40 (1%)	184 (5%)
Ticlopidine under any circumstances (clopidogrel and prasugrel have similar efficacy, stronger evidence and fewer side effects).	40 (1%)	NA
Antiplatelet agents with vitamin K antagonists, direct thrombin inhibitors or factor Xa inhibitors in patients with stable coronary artery, cerebrovascular or peripheral disease without a clear indication for anticoagulant therapy (no additional benefit from dual therapy).	38 (1%)	NA
Neuroleptics (they can cause gait dyspraxia and parkinsonism increasing the risk of falling).	30 (1%)	19 (1%)
NSAIDs in patients with known hypertension (risk of exacerbation of hypertension) or heart failure (risk of exacerbation of heart failure).	NA	150 (4%)
Central-acting antihypertensives (methyldopa, clonidine, moxonidine, rilmenidine, guanfacine), unless there is evidence of intolerance or lack of efficacy with other classes of hypertensives.	NA	78 (2%)
Benzodiazepines in patients with acute or chronic respiratory failure (risk of exacerbation of respiratory failure).	NA	70 (2%)
Oral or transdermal opioids as first-line therapy for mild pain (not indicated by the WHO analgesic scale).	NA	42 (1%)
Anti-muscarinic bronchodilators (hypatropium, tiotropium) with a history of glaucoma (they can exacerbate glaucoma).	NA	33 (1%)
Non-benzodiazepine hypnotics (they can cause prolonged daytime sedation and ataxia, increasing the risk of falling).	NA	20 (1%)
NSAIDs if GFR <50 ml/min/1.73 m2 (risk of deterioration of renal function).	NA	19 (1%)
Other PIMs that individually were not quantitatively relevant.	114 (4%)	131 (4%)
**PPOs detected using the START criteria**	**n = 2729**	**n = 3305**
Antiplatelet therapy (aspirin or clopidogrel or prasugrel or ticagrelor) with a documented history of coronary, cerebral or peripheral vascular disease.	606 (22%)	499 (15%)
Statins in patients with a documented history of coronary, cerebral or peripheral vascular disease, unless the patient is dying or> 85 years of age.	562 (21%)	491 (15%)
β-blockers (bisoprolol, nebivolol, metoprolol or carvedilol) in patients with stable systolic heart failure.	560 (21%)	200 (6%)
ACEIs in patients with systolic heart failure and/or documented coronary heart disease.	354 (13%)	359 (11%)
α1-antagonists in patients with symptomatic prostatism.	168 (6%)	131 (4%)
Xanthine oxidase inhibitors (allopurinol, febuxostat) in patients with a history of recurrent episodes of gout.	118 (4%)	104 (3%)
β2-inhaled agonists or antimuscarinic bronchodilators (ipratropium, tiotropium) for mild or moderate asthma or COPD.	65 (2%)	598 (18%)
5-alpha reductase inhibitors in patients with symptomatic prostatism.	63 (2%)	42 (1%)
Inhaled corticosteroids for moderate asthma or COPD.	59 (2%)	335 (10%)
Vitamin D supplement in older people who are at home or who have falls or osteopenia (bone mineral density T-score> -1.0 but <-2.5 at multiple sites).	49 (2%)	NA
SSRIs (or SNRIs or Pregabalin if SSRIs are contraindicated) for persistent severe anxiety.	38 (1%)	NA
Bone therapy (bisphosphonate, strontium ranelate, teriparatide, denosumab) in patients with documented osteoporosis, in which there is no pharmacological or clinical status contraindication (T-score of bone mineral density> -2.5 in multiple sites) and/or previous history of fragility fractures.	27 (1%)	NA
Laxatives in patients who regularly take opioids.	NA	265 (8%)
Aspirin (75–160 mg once daily) in presence of chronic atrial fibrillation, in patients in whom vitamin K antagonists or direct thrombin inhibitors or factor Xa inhibitors are contraindicated.	NA	146 (4%)
β-blockers in patients with ischemic heart disease.	NA	48 (1%)
ACEIs or ARBs in diabetes with evidence of kidney disease.	NA	27 (1%)
Other PPOs that individually were not quantitatively relevant.	60 (2%)	60 (2%)

The wording NA (Not Applicable) corresponds to PIMs or PPOs detected at admission but not at hospitalization, or vice versa.

The application of the START criteria has shown an increase of the percentage of patients with at least one PPO, from 72% at admission to 77% at the hospital stay. This increase is probably due to the increase in prescriptions of inhaled β2-agonists or antimuscarinics alone or in association with corticosteroids for asthma or Chronic Obstructive Pulmonary Disease (COPD).

The comparison resulting from the application of the IPET criteria at admission and at hospital stay revealed an increasing trend of patient with PIMs, from 11% on admission to 17% on admission. 44% of the detected PIMs during hospital stay were related to the prescription of NSAIDs in patients with high blood pressure (Table 6).

**Table 6:** PIMs detected with IPET criteria both at admission and hospitalization.

PIMs detected using the IPET criteria	Admission	Hospital stay
**Total of detected PIMs**	**n = 205**	**n = 273**
β-blockers in patients with COPD.	6 (3%)	44 (16%)
β-blockers in patients with congestive heart failure.	22 (11%)	35 (13%)
Ca-antagonists (except amlodipine and felodipine) in patients with congestive heart failure.	151 (74%)	63 (23%)
Thiazide diuretics in patients with gout.	NA	2 (1%)
Long-acting benzodiazepines (chlordiazepoxide, chlorazepate, diazepam, flurazepam, clonazepam, nitrazepam).	6 (3%)	4 (1%)
Tricyclic antidepressants in patients with glaucoma.	NA	NA
Tricyclic antidepressants in patients with heart block.	NA	1 (1%)
Tricyclic antidepressants in patients with active metabolites (imipramine, doxepine, amitriptyline).	11 (5%)	NA
Methylphenidate for depression.	NA	NA
NSAIDs in patients with peptic ulcer.	NA	3 (1%)
NSAIDs in patients with hypertension.	9 (4%)	121 (44%)
Long-term treatment with NSAIDs for osteoarthritis.	NA	NA
Anticholinergic drugs to treat the adverse effects of antipsychotic drugs.	NA	NA
Long-term use of difenoxylate for the treatment of diarrhea.	NA	NA

The wording NA (Not Applicable) corresponds to PIMs or PPOs detected at admission but not at hospitalization, or vice versa.

The statistical analysis, carried out through Pearson's correlation *r* of the index with the comorbidities, showed a significant correlation between the comorbidities and the index of PIMs and PPOs at hospital stay. At admission, however, there was no significant correlation between the index relating to the Beers and IPET criteria and the comorbidities (Table 7).

**Table 7:** Number of outliers and Pearson correlation (*r*) between the index of each criteria and the comorbidities detected both at admission and at hospitalization.

	Admission			Hospital stay		
	outliers	*r*	*p*	outliers	*r*	*p*
Beers	n = 21	-0.036	0.130	n = 27	0.218	< 0.0001
STOPP	n = 20	-0.118	< 0.0001	n = 21	0.076	0.004
START	n = 1	0.273	< 0.0001	n = 19	0.377	< 0.0001
IPET	n = 34	0.038	0.103	n = 23	0.137	< 0.0001

From the comparison of the average index relating to each criterion, it was lower at the hospital stay than at admission (Table 3) for all criteria due to the fact that, as can be seen from Table 1, the average of the drugs registered at admission was approximately half that of the hospital stay. The calculated Coehn’s *d* was of medium level for the STOPP and IPET criteria (respectively -0.488 and -0.401) and of small level for the Beers and START criteria (respectively -0.124 and -0.092). The two-tailed Student's *t* test carried out showed significant differences emerged as regards the Beers criteria (*t* = 3,353, *df* = 3258, *p* < 0.0001), STOPP (*t* = 13.30, *df* = 3258, *p* < 0.0001) and START (*t* = 10.88, *df* = 3258, *p* < 0.0001). However, the differences were not significant for the IPET criteria (*t* = 1,908, *df* = 3258, *p* < 0.0001). This could be due to the fact that the latter consist of 14 items and therefore are less frequently detected than the other criteria consisting of many more items (i.e. 260 Beers, 75 STOPP and 33 START).

## Discussion

The analysis of geriatric therapies carried out at admission and at hospitalization showed an increase in the average consumption of drugs during the hospital stay. This propensity for polytherapy is attributable to differences often due to the changed clinical conditions of the patient during his hospitalization. In this context, the correct implementation of the medication reconciliation in the transition of patients between care settings represents an essential tool for making safe and appropriate prescriptive decisions [16].

PPIs were the most prescribed PIMs according to Beers criteria in both cohorts of patients. In fact, prolonged use of these drugs has been associated with a higher risk of osteopenia and osteoporosis, with consequent predisposition to bone fractures [17–19]. This significant association between proton pump inhibitors for protracted periods and osteopenia is probably due to impaired absorption of calcium, vitamin B12, iron and magnesium [20–22] which can change bone mineral density. Prolonged use of proton pump inhibitors can also increase the risk of Clostridium difficile infections [23]. Therefore, Beers' criteria recommend avoiding its use for more than 8 weeks, except in high-risk patients, such as patients with erosive esophagitis, Barret's esophagitis, pathological hypersecretion, ongoing therapy with corticosteroids or oral NSAIDs, or therapeutic failure of H2-antagonists. The Beers criteria also recommend minimizing the number of CNS active drugs (such as opioids, benzodiazepines and non-benzodiazepine hypnotics, antidepressants and antipsychotics) to a maximum of 3 [24], especially in elderly patients at risk of falling and fractures. This aspect is particularly important in the hospital setting, considering that falls in hospitalized patients represent the most common adverse event and can lead to an increase in hospital stay and additional diagnostic and therapeutic activities. The Standards of the Joint Commission International, of which ISMETT has been an accredited structure since 2009, also pay particular attention to the evaluation of the risk of falling for patients and a possible revaluation in case of modification of the health conditions during hospitalization [25].

PPIs administered for more than 8 weeks, detected as main PIMs according to the STOPP criteria at admission, were not detected with the same occurrence at the hospital stay, probably because the percentage of inpatients at ISMETT for more than 31 days was relatively low (Table 1). Vasodilators (α1-antagonists, Ca-antagonists, long-acting nitrates, ACE inhibitors, sartans, diazoxide, minoxidil, hydralazine) were, instead, the most detected PIMs according to the STOPP criteria during hospital stay in patients with persistent postural hypotension, as they can predispose to risk of syncope and falls. In this regard, the 2018 guidelines of the European Society of Cardiology for the diagnosis and treatment of syncope suggest that the reduction or suspension of hypotensive therapy aimed at a systolic pressure of 140 mmHg is effective in reducing syncopal recurrence in predisposed patients [26]. The other frequently reported PIM with the STOPP criteria during hospitalization was related to drugs causing constipation (anticholinergics, oral iron, opioids, verapamil, aluminum-based antacids) in patients with chronic constipation, with consequent risk of exacerbation of constipation. In this regard, during hospitalization, the percentage of PIM referring to the prescription of opioids without concomitant laxative therapy also increased. This aspect is very important considering that chronic constipation is a very common pathophysiological phenomenon in elderly patients, especially women, due to the slowing of intestinal transit, the reduction of sensorimotor functions and the atrophy of the anal sphincters. Therefore, the prevention of iatrogenic constipation is fundamental [27]. Regarding the START criteria, the most detected PPOs were the β2-agonists or antimuscarinics inhaled alone or in combination with corticosteroids for asthma or COPD. In this regard, the most recent revision of the Global Initiative for Chronic Obstructive Lung Disease (GOLD 2019) [28] reaffirms that the inhaled administration of bronchodilators is recommended over the oral route, and that associations of bronchodilators with different mechanism and duration of action can improve the degree of bronchodilation, with a lower risk of adverse events than the dosage increase of a single bronchodilator [29–31]. The main PIM found with the IPET criteria during hospitalization was the prescription of NSAIDs in patients with high blood pressure. In general, they can increase blood pressure due to the inhibition of the synthesis of prostaglandins, which leads to a direct effect in modulating renal blood flow [32]. In addition, NSAIDs can cause an increase in serum aldosterone, with consequent sodium retention and hypertensive effect [33].

The statistical analysis showed a significant correlation between the comorbidities and the index of prescriptive inappropriateness and appropriateness at the hospital stay. Therefore, as shown by the descriptive statistics, during the hospital stay, the impact of comorbidities on polytherapy and, consequently, also on the index relating to the criteria is significant. On admission, however, there was no significant correlation between the index relating to the Beers and IPET criteria and the comorbidities. These results are probably due to the fact that upon admission the prescribed therapies are greatly influenced by the variability conferred by the numerous general physicians who have taken care of the elderly patients.

## Conclusion

The activity carried out in this retrospective study has highlighted the main PIMs and PPOs in admitted and hospitalized patients at our Institute. A propensity to polytherapy emerged during hospital stay. The prescriptive inappropriate criteria were a useful tool for assessing the quality of prescriptions in the geriatric population and identifying their critical issues. An evidence-based approach based on the application of these criteria can allow to raise the standards of care and improve decision making in geriatric prescriptions. To make physicians aware of the use of these tools and to improve care for the elderly patients an educational brochure has been created. Furthermore, the management of drug therapy in the elderly patient requires a multidisciplinary approach, in which the TP can support physicians in choosing the safest and most appropriate therapies for elderly patients. A possible limitation of our study, which will be implemented later, is that two different cohorts of elderly patients were analyzed at admission and hospitalization, the advantage is represented by the sample size. Furthermore, the quality of pharmacological prescriptions could also be assessed at discharge as a tool to prevent further hospital readmissions following drug-related events [34]. This would increase the safety and appropriateness of the drugs prescribed in all transitions of care (admission, hospital stay and discharge). Other strategies to be implemented could be the reduction of the number of taken drugs through the support algorithms for de-description [35] or the use of non-pharmacological approaches to treat common diseases of elderly patient [36,37].
